# Selective and Wash‐Resistant Fluorescent Dihydrocodeinone Derivatives Allow Single‐Molecule Imaging of μ‐Opioid Receptor Dimerization

**DOI:** 10.1002/anie.201912683

**Published:** 2020-01-07

**Authors:** Christian Gentzsch, Kerstin Seier, Antonios Drakopoulos, Marie‐Lise Jobin, Yann Lanoiselée, Zsombor Koszegi, Damien Maurel, Rémy Sounier, Harald Hübner, Peter Gmeiner, Sébastien Granier, Davide Calebiro, Michael Decker

**Affiliations:** ^1^ Pharmaceutical and Medicinal Chemistry Institute of Pharmacy and Food Chemistry Julius Maximilian University of Würzburg Am Hubland 97074 Würzburg Germany; ^2^ Institute of Pharmacology and Toxicology Julius Maximilian University of Würzburg Versbacher Strasse 9 97078 Würzburg Germany; ^3^ Institute of Metabolism and Systems Research & Centre of Membrane Proteins and Receptors University of Birmingham IBR Tower, Level 2, Edgbaston Birmingham B152TT UK; ^4^ ARPEGE (Pharmacology Screening Interactome) platform facility Institut de Génomique Fonctionnelle Université de Montpellier, CNRS, INSERM 141, rue de la Cardonille 34094 Montpellier Cedex 05 France; ^5^ Institut de Génomique Fonctionnelle Université de Montpellier, CNRS, INSERM 141, rue de la Cardonille 34094 Montpellier Cedex 05 France; ^6^ Medicinal Chemistry Department of Chemistry and Pharmacy Friedrich-Alexander University of Erlangen-Nuremberg 91058 Erlangen Germany

**Keywords:** fluorescent probes, G-protein coupled receptor, homodimerization, opioid ligands, single-molecule microscopy

## Abstract

μ‐Opioid receptors (μ‐ORs) play a critical role in the modulation of pain and mediate the effects of the most powerful analgesic drugs. Despite extensive efforts, it remains insufficiently understood how μ‐ORs produce specific effects in living cells. We developed new fluorescent ligands based on the μ‐OR antagonist *E‐p*‐nitrocinnamoylamino‐dihydrocodeinone (CACO), that display high affinity, long residence time and pronounced selectivity. Using these ligands, we achieved single‐molecule imaging of μ‐ORs on the surface of living cells at physiological expression levels. Our results reveal a high heterogeneity in the diffusion of μ‐ORs, with a relevant immobile fraction. Using a pair of fluorescent ligands of different color, we provide evidence that μ‐ORs interact with each other to form short‐lived homodimers on the plasma membrane. This approach provides a new strategy to investigate μ‐OR pharmacology at single‐molecule level.

## Graphical abstract

**A pair of fluorescent**, selective μ‐opioid receptor‐ligands was synthesized and applied in single‐molecule microscopy experiments. After their pharmacological characterization, the ligands were used to investigate diffusion behavior and homodimerization of wild‐type μ‐opioid receptors on the surface of living cells.

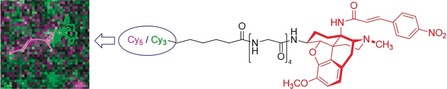

## Introduction

Opioid receptors (ORs) belong to the family A of rhodopsin‐like G protein‐coupled receptors and occur in three major subtypes, μ, δ and κ.^1^ They are predominantly found in the central and peripheral nervous system, where they modulate transmission and perception of pain. Importantly, μ‐ORs mediate most therapeutic effects of opioids, which are the most powerful, but also most addictive analgesics known to date.^2^ In contrast, κ‐ and δ‐OR activation causes weaker analgesia, but has been associated with less side effects.^3^ The effects of opioids on neurons are mainly mediated by activation of heterotrimeric G_i_ proteins, which inhibit cAMP production, while opening G protein‐coupled inward rectifying potassium channels (GIRK) and closing voltage‐dependent N‐type Ca^2+^ channels via G_βγ_ subunits.^2^, ^3^

Because of their importance as drug targets, μ‐ORs have been intensively investigated both, in vitro and in vivo.^1^, ^2^, ^3^, ^4^ A major breakthrough in the field has been the determination of high‐resolution three‐dimensional structures of the μ‐OR in complex with an antagonist and, later on, with an agonist and G protein mimetic nanobodies, as well as most recently by cryo‐electron microscopy.^5^ These studies have provided important insights into the mechanisms of ligand binding and receptor activation, which might pave the way to the rational design of new analgesics with improved efficacy and less side effects, such as addiction. Interestingly, in the above studies μ‐ORs were found to crystallize as dimers.5a, 5b Largely based on previous experiments applying resonance energy transfer methods, these findings support the hypothesis that μ‐ORs might form homodimers.^1^, 4b, 6 However, the nanoscale organization and dynamics of μ‐ORs on the surface of living cells remain largely unknown, mostly due to technical limitations of conventional methods. These typically include the requirement of cell disruption, insufficient spatiotemporal resolution and/or averaging over thousands or even millions of receptors.^7^

In an attempt to overcome such limitations, we and others have developed innovative methods based on single‐molecule microscopy, which allow imaging of individual receptors and other molecules on the surface of living cells with a temporal resolution of approximately 28.4 ms and a spatial resolution of approximately 20 nm, which is at least 10 times below the best theoretical resolution of conventional fluorescence microscopy.^8^ This approach can provide a highly quantitative characterization of dynamic events, such as protein‐protein interactions among membrane receptors,^9^ even when involving only a small fraction of the investigated molecules.9a, 9c, 10

Here, we present the synthesis and single‐molecule microscopy application of new fluorescent, selective μ‐OR ligands to study unmodified receptors in living cells at physiological expression levels.

## Results and Discussion

**Figure 1:**
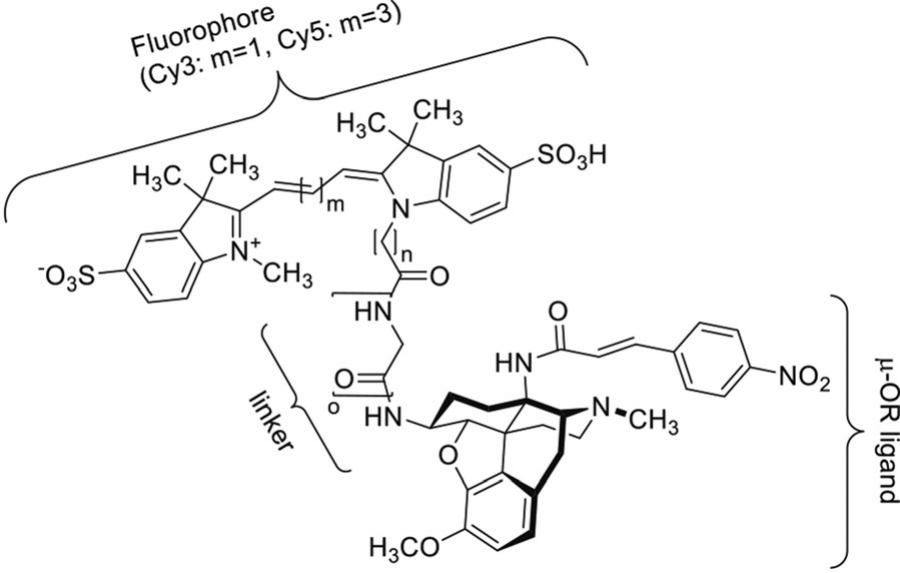
Structure of the fluorescent, selective μ‐opioid receptor ligands (*n*=5, *o*=4).

The structure of the ligands consists of a pharmacologically active compound, a fluorophore, and a linker (Figure 1). All three units have been chosen with regard to their individual and specific characteristics to achieve high affinity and selectivity as well as optimal optical properties for single‐molecule fluorescence microscopy, for example, high signal‐to‐noise ratios and low blinking and bleaching rates.

The pharmacologically active moiety (parent ligand) is based upon *E‐p*‐nitrocinnamoylamino‐dihydrocodeinone (CACO), which had been described to be a potent and selective μ‐OR ligand, and to retain such properties upon conjugation with the small organic fluorophore BODIPY (EC_50_=24.4 nm, δ/κ>1000 nm) through a small alkylene linker.^11^ The unlabeled ligand has been described to act as short‐term agonist and long‐term antagonist with an IC_50_ value of 0.46±0.003 nm for μ‐OR (*δ*=4.2±1.3 nm, *κ*=19±2.8 nm). A *K*_d_ of 0.52±0.14 nm was measured by means of saturation binding experiments with [^3^H]DAMGO.^12^ The nitrocinnamoyl part of the active compound contains a Michael acceptor, which potentially forms covalent bonds with nucleophilic side‐chains of amino acids in the μ‐OR binding pocket. This has been shown in the μ‐OR crystal structure for β‐funaltrexamine.5a

The pentylene linker connects the pharmacologically active moiety and the fluorophore. In our probe design, we incorporated a tetraglycine into the linker, inspired by previous studies based on GPCR‐imaging with fluorescent ligands, which yielded improved results.^13^ Cyanine 3 and 5 (Cy3/5) were chosen as fluorophores because of their advantageous optical properties which make them particularly suited for single‐molecule microscopy, including emission in the red and near infrared regions of the spectrum, respectively, as well as high absorption coefficients and quantum yields.^14^

**Scheme 1:**
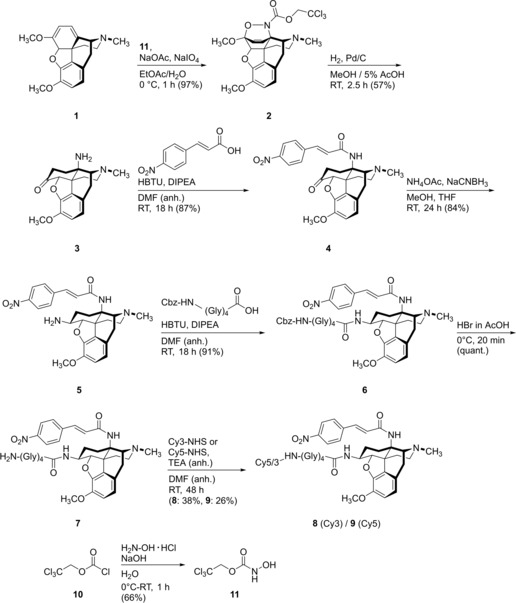
Synthesis of the fluorescent, μ‐OR selective ligands **8** and **9**.

The synthesis shown in Scheme 1 started with a hetero‐Diels–Alder reaction of thebaine **1** and the intermediately oxidized *N*‐hydroxycarbamate **11**, prepared from the respective chloroformiate **10**.^15^ The resulting cycloadduct **2** was hydrogenated to give dihydrocodeinone **3**.^16^ A nitrocinnamoyl moiety was introduced by coupling the C14‐amino group of **3** with the respective activated acid, yielding compound **4**.^17^ Subsequent reductive amination of the C‐6‐keto group led to compound **5**.^11^ In a preliminary approach, we directly used the NHS‐activated dyes to couple them to the newly introduced amino‐group. However, these probes had shown high background and a poor signal‐to‐noise ratio in single‐molecule microscopy experiments (see Supporting Information). While an alkylene chain might be sufficient to bridge pharmacophore and fluorophore and define the distance between the bulky residues, the tetraglycine moiety additionally increases the polarity of the compound and prevents sticking to the plasma membrane (Figure 1). This led to a major increase in selectivity and reduction of background. The newly introduced amino group of **5** was coupled to *N*‐Cbz‐protected tetraglycine, yielding compound **6**.^17^ After deprotection of Cbz with hydrobromic acid, the free amine **7** was coupled to the NHS‐activated dyes Cy3 and Cy5 to give the desired fluorescent ligands **8** and **9** (Scheme 1).

**Figure 2:**
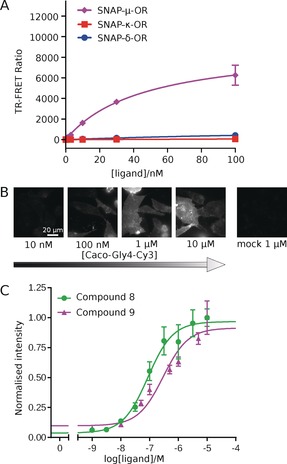
Characterization of compounds **8** and **9**. A) Selectivity of compound **9** for μ‐OR versus κ‐OR and δ‐OR. HEK293 cells were transfected with either OR subtype carrying a SNAP‐tag at its N‐terminus and labeled with SNAP‐Lumi4‐Tb. Binding of the fluorescent ligand was detected via HTRF. The mean ± S.E.M. of three independent experiments is shown. B) Representative TIRF images of CHO cells either transfected with wild‐type μ‐OR or mock transfected and treated with increasing concentrations of compound **8**. C. Concentration‐binding relationships for compounds **8** and **9** obtained from images as in B. The mean ± S.E.M. of three independent experiments is shown (32 to 86 cells per data point).

Saturation binding curves obtained for compound **9** showed its selectivity for μ‐OR (Figure 2 A) in a homogenous time‐resolved FRET (HTRF) assay with HEK293 cells, which gave optimal results. No significant binding was observed to the other OR‐subtypes (Figure 2 A). These findings are in good agreement with previous binding studies on the parent compound CACO.^12^

**Figure 3:**
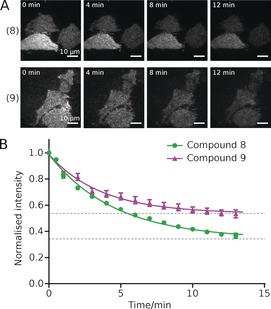
Wash resistance of compounds **8** and **9**. A) Representative TIRF images of CHO cells transiently transfected with wild‐type μ‐OR and treated with 1 μm of compound **8** (top) or **9** (bottom) for 20 min followed by washing. The same cells were imaged every minute during the wash. B) Plots of compound **8**/**9** dissociation over time obtained from image sequences as in (A). Photobleaching of compounds **8** during this time was negligible; the intensity values of compound **9** were corrected for photobleaching. The mean ± S.E.M. of four independent experiments is shown (13 and 15 cells per data point, respectively). Data were fitted with a one phase exponential decay plus a constant.

We then tested compounds **8** (Cy3 conjugate) and **9** (Cy5 conjugate) against wild‐type μ‐OR expressed in CHO cells, which adhere very well to glass‐coverslips, resulting in a particularly flat plasma membrane. Compounds **8** and **9** bound to cell‐surface receptors were selectively imaged by total internal reflection fluorescence microscopy (TIRF), which illuminates only approximately 200 nm at the interface between the coverslip and the cells (Figure 2 B). CHO cells devoid of μ‐OR expression were used as control for unspecific binding. The results confirmed a highly specific binding of the compounds to μ‐ORs, with negligible unspecific binding to the cells or the coverslip (Figure 2 B). This approach also allowed us to obtain concentration‐binding relationships for both compounds. In these experiments, we incubated CHO cells with increasing concentrations of either compound **8** or **9** for 20 min and imaged them by TIRF microscopy. The mean intensities of at least 40 cells per condition were averaged for each concentration of ligand, which allowed us to estimate their affinities (*K*_D_) (Figure 2 C). A *K*_D_ value of 87±49 nm for compound **8** was reached, whereas compound **9** showed a three‐fold lower affinity for μ‐ORs with an estimated *K*_D_ value of 295±141 nm. The initial affinity of CACO for the μ‐OR was reported to be 0.52±0.14 nm.^12^ Intrinsic activity of compound **8** was determined in an inositol mono phosphate‐accumulation assay for G‐protein mediated signaling. In this assay, the compound acted as a partial agonist (EC_50_=190 nm, *E*_max_=57 % of maximal response to morphine), while it was inactive in the β‐arrestin‐2 recruitment assay (cf. Supporting Information). Although chemical modifications often lead to changes in the pharmacological profiles of small molecules, both compounds retain high affinity towards μ‐OR. A 50 % wash resistance of CACO has been reported in competition binding experiments with DAMGO, probably due to the capability of the 14β‐*p*‐nitrocinnamoylamino‐side chain of CACO to bind covalently to the μ‐OR.^12^ Fluorescent probes for single‐molecule microscopy not only need to possess high labeling efficiency, but ideally, also long residence time on the receptor. Thus, intrigued by the possibility that CACO may bind covalently to μ‐OR, we investigated if compounds **8** and **9** also retained a high wash resistance. For this purpose, we performed washing experiments in CHO cells transiently treated with either compound **8** or **9** for 20 min and imaged by TIRF microscopy (Figure 3). During washing, we observed a slow decrease of fluorescence intensity, until it reached a plateau at approximately 33 % and 53 % of the initial values for compound **8** and **9**, respectively. Importantly, these results indicate that both fluorescent ligands exhibit a long residence time on μ‐OR, with a fraction of virtually non‐dissociating receptors.

**Figure 4:**
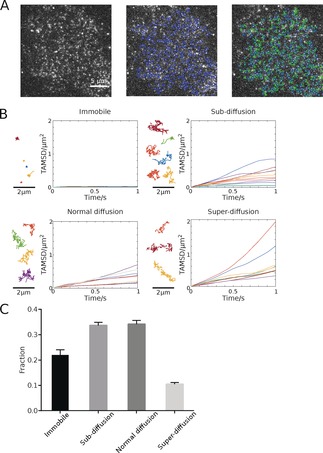
Single‐molecule imaging of μ‐ORs on the surface of living cells using compound **8**. A) Representative results. A frame of a representative TIRF image sequence (left) and the results of the automated single‐particle detection (middle) and tracking algorithm used to follow individual μ‐ORs (right) is shown. The current position of individual receptors (blue circles) and their trajectories (green splines) are shown. B) Time‐averaged mean square displacement (TAMSD) analysis on the obtained μ‐OR trajectories. Based on the results, the trajectories were classified into four categories: virtually immobile, sub‐diffusive, normal diffusive and super‐diffusive. Shown are examples of trajectories belonging to the four categories and their corresponding TAMSD curves. C) Distribution of μ‐ORs trajectories in the four categories identified by the TAMSD analysis. Data are mean ± S.E.M of 29 individual cells (2,225 trajectories).

Next, we tested the applicability of the new fluorescent ligands for single‐molecule fluorescence microscopy. Both compounds showed excellent photophysical properties, giving rise to easily detectable fluorescent spots in TIRF microscopy. Compound **8** showed slower photobleaching in comparison to compound **9**, consistent with the known photophysical properties of Cy5 and Cy3, respectively.^18^ Therefore, the Cy3 ligand was used for subsequent single‐color experiments. CHO cells transiently transfected to express wild‐type μ‐OR at low physiological densities (approximately 0.8 receptors per μm^2^) were incubated with a saturating concentration of compound **8** (1 μm) and imaged by fast TIRF microscopy. The transfected cells were easily distinguishable from the background, confirming a highly specific binding (Figure 4 A). Individual μ‐ORs carrying a fluorescent ligand were detected and tracked using an automated algorithm.9a, 9c, 19 A time‐averaged mean square displacement (TAMSD) analysis was performed, which allowed investigating the diffusion of the receptors within the plasma membrane (cf. Supporting Information). This analysis revealed a high heterogeneity in the mobility of μ‐ORs on the plasma membrane. Individual μ‐OR particles were classified into four categories based on the type and speed of their motion.9c A percentage of 22±2 % of the receptors were virtually immobile, likely due to trapping in small nanodomains or binding to immobile membrane structures. Sub‐diffusive motion was observed for 34±1 %, meaning their motion was hindered by either crowding or interactions with their environment. This phenomenon has been previously described for other membrane receptors and can arise from different factors, such as transient trapping in nanodomains.9c, 20

Another 34±1 % of the receptors showed normal diffusion, that is, Brownian motion. Super‐diffusion, that is, directional motion, was observed for the remaining 10±1 % of particles.

These results are in agreement with previous findings for other prototypical GPCRs like the β_2_‐ or the α_2A_‐adrenergic receptor.9c

**Figure 5:**
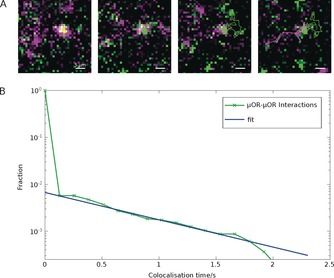
Single‐molecule imaging of transient μ‐OR interactions. A) Example of a transient co‐localization (yellow) observed between two μ‐ORs, one labeled with compound **8** (green) and the other with compound **9** (magenta). Both receptors interrupted their motion during the co‐localization event. B) Estimation of the frequency and duration of interactions between μ‐ORs based on deconvolution of their co‐localization times with those obtained between μ‐ORs and CD86, where no interactions are expected. Data are from 51832 co‐localizations obtained from 25 individual cells for μ‐OR‐μ‐OR and 61259 co‐localizations for CD86‐μ‐OR.

We then explored the applicability of compounds **8** and **9** in single‐molecule microscopy experiments to investigate the dimerization of μ‐OR at low/physiological expression levels in living cells. For this purpose, CHO cells were transiently transfected with wild‐type μ‐OR, yielding a total receptor density of around 1.7 particles per μm^2^. The cells were then simultaneously labeled with a mixture of compounds **8** and **9** (1 μm and 0.5 μm, respectively) to label as many receptors as possible (approximately 80 %) with either compound, while keeping unspecific binding to the glass‐coverslip low. Then, imaging by fast two‐color TIRF microscopy was carried out. The co‐localization between receptors labeled with compound **8** and **9** was analyzed by automated computer algorithms as previously described.9c In order to correct for the presence of random co‐localizations and to estimate the duration of μ‐OR interactions, we applied a previously developed method based on deconvolution of the co‐localization times.9c For this purpose, we additionally performed the same experiment using μ‐ORs labeled with compound **8** and CD86, a monomeric control protein not interacting with μ‐OR, labeled with Alexa Fluor 647 via a SNAP‐tag fused at its N‐terminus (Figure S2 in the Supporting Information).9a This served as control for the co‐localizations expected in the absence of interactions. The deconvolution analysis revealed that more than 95 % of μ‐ORs were diffusing on the plasma membrane as monomers. However, it also revealed a small but consistent fraction of receptors that apparently underwent transient interactions lasting approximately 1–2 seconds. At the low receptor densities analyzed, this fraction of μ‐ORs that were interacting at any given time was approximately 4–5 %. Although this represents only a fraction of the receptors, this value is remarkably similar to the one observed between active receptors and G proteins,9c a fundamental interaction in GPCR signaling, suggesting that although involving only a small fraction of receptors it might nevertheless be biologically relevant. Even though the presence of an even smaller fraction of higher order oligomers cannot be completely ruled out, the intensities of the majority of receptor particles and their bleaching behavior (i.e., number of observed photobleaching steps) were consistent with them being monomers or at most dimers. By deconvolving the distribution of the co‐localization times observed between μ‐ORs labeled with compounds **8** and **9,** and those obtained between μ‐OR and CD86, we were able to estimate the frequency and duration of the interactions between μ‐ORs. The resulting relaxation plot of μ‐OR interactions (Figure 5 B) indicated that μ‐ORs were dissociating following an exponential decay, with an estimated time constant (*τ*) of 1.797±0.487 s (corresponding to a dissociation rate constant, *k*_off_, of 0.557±0.207 s^−1^). This value is in good agreement with previous results obtained with prototypical family A GPCRs.9a–9c, 21 This approach also allowed us to calculate the two‐dimensional association rate (*k*_on_) for interactions between μ‐ORs, that is, the formation of dimers, which we estimated to be 0.020±0.004 μm^2^ molecule^−1^ s^−1^. The estimated *k*_on_ and *k*_off_ values give a dissociation equilibrium constant (*K*_d_) of 27.43±11.75 molecules per μm^2^, allowing to predict the fraction of μ‐ORs in monomeric or dimeric state depending on their density on the plasma membrane. Based on the obtained *K*_d_ value, we estimate that a μ‐OR density of approximately 27 molecules per μm^2^ would be required for 50 % of them to be in dimeric state (approximately 7 dimers and 14 monomers per μm^2^). Since such densities might occur at synapses,^22^ it is possible that a relevant fraction of μ‐ORs might form transient dimers in vivo.^23^

**Figure 6:**
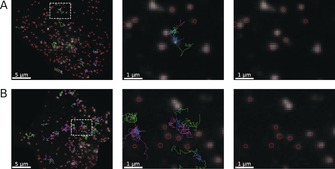
Single‐molecule imaging of transient μ‐OR interactions in relationship to the location of clathrin coated pits (CCPs). CHO cells were transiently transfected with wild‐type μ‐OR and GFP‐clathrin. The cells were treated with compound **8** (1 μm) and compound **9** (0.5 μm) for 20 min. After one rapid wash, the cells were imaged by fast two‐color TIRF microscopy to track the receptors, followed by acquisition of a CCP image. Shown are images of a representative cell (left) with the obtained trajectories of receptors (green and magenta, respectively) undergoing interactions (blue) overlaid on the CCP image (grey). Red circles correspond to individual CCPs. Middle, enlarged views of the regions delimited by the dashed boxes. Right, same regions without trajectories. A) interactions occurring within or near CCPs. B) interactions occurring outside CCPs.

Interestingly, the majority of receptors seemed to transiently stop diffusing while interacting (Figure 5 A). A possible explanation for this is that the observed interactions might represent receptors being simultaneously recruited to the same clathrin‐coated pit (CCP) before internalization. To investigate this possibility, we repeated the same experiment in the presence of co‐transfected GFP‐tagged clathrin (Figure 6). Computational analyses showed that 77±9 % of all observed μ‐OR interactions happen outside of CCPs, with only 23±9 % occurring within or near CCPs. These results suggest that other mechanisms are involved in the observed transient trapping of μ‐ORs during their interactions.

## Conclusion

Whereas GPCRs have long been believed to be solely monomeric receptors, a growing body of evidence obtained mainly using fluorescence and bioluminescence resonance energy transfer (FRET and BRET) suggests that they might form dimers or even higher order oligomers.^7^, 10b, 24 However, the occurrence of GPCR dimerization at physiological expression levels and their stability remain—with few notable exceptions such as family C GPCRs—controversial. These debates also extend to μ‐OR, for which contrasting results have been obtained using methods relying on different expression levels, ranging from physiological levels to overexpression and even purification. Whereas several of the published studies provide evidence in favor of dimerization,^1^, 4b, 5a, 25 others indicate that μ‐ORs can function as monomers or the fraction of dimers is negligible.^26^ Our data, obtained on wild‐type receptors expressed at levels in living cells contribute to resolve this controversy by showing that whereas most μ‐ORs are monomeric, a small, though potentially biologically relevant, fraction of receptors undergo transient dimerization. As the receptors appear to be in equilibrium between monomers and dimers, higher level of dimerization might be achieved at sites of particular high receptor density such as synapses.

In summary, we developed two novel, fluorescent, subtype‐selective ligands for the μ‐OR with binding affinities in the nanomolar range and the advantageous property of wash resistance. Importantly, we show that the favorable properties of these ligands make them ideal for single‐molecule microscopy. We used this set of ligands to investigate the diffusion behavior of μ‐ORs as well as their interactions at physiological expression levels. Our results reveal the occurrence of transient receptor interactions, consistent with a dynamic equilibrium between monomers and dimers. As only a small fraction of receptors was found to be interacting with each other in our study, which is too small to be detected in ensemble measurements, our results may help to reconcile some of the apparent discrepancies between previous studies. Importantly, our results reveal dynamic interactions among μ‐ORs that occur outside CCPs that would have likely been missed using ensemble methods. This new approach opens up new venues to investigate μ‐OR biology and pharmacology in living cells with unprecedented spatiotemporal resolution.

## Experimental Section

Comprehensive details of the experimental design can be found in the Supporting Information.

## Conflict of interest

The authors declare no conflict of interest.

## Supporting information

As a service to our authors and readers, this journal provides supporting information supplied by the authors. Such materials are peer reviewed and may be re‐organized for online delivery, but are not copy‐edited or typeset. Technical support issues arising from supporting information (other than missing files) should be addressed to the authors.

Supplementary
