# Supplementary material for: Selective and Wash‐Resistant Fluorescent Dihydrocodeinone Derivatives Allow Single‐Molecule Imaging of μ‐Opioid Receptor Dimerization
Source: Angew Chem Int Ed Engl. 2020 Jan 7;59(15):5958–64. doi: 10.1002/anie.201912683 (PMC7125027; doi:10.1002/anie.201912683)
Supplement: Supplementary file 1 — Supplementary [file ANIE-59-5958-s001.pdf]

Angewandte Chemie
Eine Zeitschrift der Gesellschaft Deutscher Chemiker

Supporting Information

Selective and Wash-Resistant Fluorescent Dihydrocodeinone Derivatives Allow Single-Molecule Imaging of μ-Opioid Receptor Dimerization

Christian Gentzsch+, Kerstin Seier+, Antonios Drakopoulos, Marie-Lise Jobin, Yann Lanoiselée, Zsombor Koszegi, Damien Maurel, Rémy Sounier, Harald Hübner, Peter Gmeiner, Sébastien Granier, Davide Calebiro, and Michael Decker**

anie_201912683_sm_miscellaneous_information.pdf

Supporting Information

1 Synthesis
1.1 Common reagents and methods

Common reagents and solvents were obtained from commercial suppliers (Aldrich, Steinheim, Germany; Merck, Darmstadt, Germany) and were used without any further purification, unless stated otherwise.
The thin-layer chromatography for reaction-control was performed on coated plates (Silica Gel 60 F254). The substances were either visualised by their fluorescence, when irradiated with UV-light (256 nm), by spray-reagents (Dragendorff's reagent) or by their discolouration in the iodine chamber.
Silica gels with grain sizes of 0.063 - 0.2 mm (company Merck, Darmstadt, Germany) were used for manual column-chromatography. The columns were packed wet. The composition of the eluents is indicated in percentage by volume.
NMR-spectra were recorded at room temperature on a Bruker AV 400 FT-NMR-Spectrometer (company Bruker Biospin, Karlsruhe, Germany) (^{1}H: 400 MHz, ^{13}C: 100 MHz). The residual protons and the ^{13}C-resonance signals of the deuterated solvents were used as internal standards. The chemical shifts δ are indicated in [ppm] and the coupling constants in [Hz]. The signal multiplicities are abbreviated as follows: s = singlet, d = doublet, t = triplet, q = quartet, dd = doublet of doublets, m = multiplet.
Analytical HPLC was performed on a Shimadzu LC20AB system equipped with a DGU-20A3R controller, and a SPD-20A UV/Vis detector. The stationary phase was a Synergi 4u Fusion-RP (150×4.6 mm) column. A MeOH/water gradient + 0.1% formic acid (phase A/ phase B) was used as mobile phase. For analytical HPLC a flow rate of 1 mL/min was used. For preparative HPLC, a Synergi 4u Fusion-RP 80A (250×10.0 mm) column was used as stationary phase and the flow rate was 2.5 mL/min.
ESI-MS spectral data were acquired on a Shimadzu LCMS-2020 single quadrupole LC-MS (Shimadzu Europe, Duisburg, Germany).

1.2 Synthesis of 2,2,2-trichloroethyl-*N*-hydroxycarbamate (11)

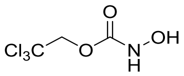

1.13 g (28.3 mmol, 6.00 eq) sodium hydroxide were dissolved in water and 1.64 g (23.6 mmol, 5.00 eq) hydroxylamine hydrochloride were added. Then 0.65 mL (4.72 mmol, 1.00 eq, 1.00 g, 1.54 g/mL) 2,2,2-trichloroethyl chloroformate were given to the solution, while vigorous stirring and cooling with ice. The reaction was stirred for 1 h at room-temperature. After that, the aqueous phase was extracted with six small portions of dichloromethane and the combined organic layers were washed with three small amounts of brine. After drying over sodium sulphate, the crude product was purified by column-chromatography (PE : EA = 2 : 1) to yield a white, crystalline solid.

Yield: 646.4 mg, 3.10 mmol, 66%.

^{1}H-NMR (400 MHz, DMSO) δ [ppm]: 4.82 (s, 2H, -CH_2-), 9.04 (brs, 1H, -OH), 10.26 (brs, 1H, -NH-).

^{13}C-NMR (100 MHz, DMSO) δ [ppm]: 73.28 (-CH_2-), 96.11 (Cl_3C-), 155.69 (C=O).

m.p.: 89°C

1.3 Synthesis of cycloadduct (2)

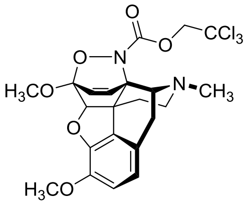

427 mg (2.05 mmol, 1.50 eq) of 2,2,2-trichloroethyl-*N*-hydroxycarbamate (11) were slowly added to a solution of 426.58 mg (1.37 mmol, 1.00 eq) Thebaine in 43 mL ethyl acetate and 438.47 mg (2.05 mmol, 1.50 eq) sodium periodate in 21.33 mL of a 0.5 M sodium acetate solution. The solution was cooled with ice and the pH-value was adjusted to 6 by addition of a few drops of 2N-HCl. The reaction was stirred for one hour at 0°C, before saturated, aqueous sodium hydrogen carbonate was added to get an alkaline reaction mixture. The ethyl acetate layer was washed with saturated, aqueous sodium thiosulphate and brine, dried over sodium sulphate and evaporated. The crude product was purified by column-chromatography (PE : EA = 3 : 1 → 2 : 1 → 1 : 1) to yield a white, crystalline solid.

Yield: 686 mg, 1.325 mmol, 97%.

^{1}H-NMR (400 MHz, $CDCl_3$) δ [ppm]: 1.16 - 2.04 (m, 2H, H-15), 2.36 - 2.72 (m, 4H, H-10 / H-16), 2.49 (s, 3H, N-CH_3), 3.42 (d, ^{3}J = 18.5 Hz, 1H, H-9), 3.63 (s, 3H, OCH_3-6), 3.81 (s, 3H, OCH_3-3), 4.59 (s, 1H, H-5), 4.91 (brs, 2H, O-CH_2), 6.06 and 6.14 (ABq, ^{3}J = 8.9 Hz, 2H, H-7 / H-8), 6.58 and 6.68 (ABq, $^{3}J_1$ = 8.18 Hz, $^{3}J_2$ = 8.19 Hz, 2H, H-1 / H-2).

^{13}C-NMR (100 MHz, $CDCl_3$) δ [ppm]: 23.79 (C-10), 33.45 (C-15), 43.23 (N-CH_3), 45.28 (C-16), 53.42 (OCH_3-6), 56.84 (OCH_3-3), 58.64 (C-9), 75.59 (O-CH_2), 87.52 (CCl_3), 90.45 (C-5), 104.90 (C-11), 114.95 (C-2), 119.95 (C-1), 123.05 (C-7 / C-8), 142.50 (C-3), 148.25 (C-4).

ESI-MS: 517.05 m/z $[M+H]^+$, calculated 516.06 m/z (100%).

m.p.: 181°C

1.4 Synthesis of 14β-amino-7,8-dihydrocodeinone (3)

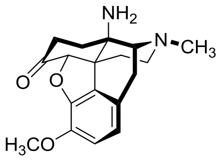

351 mg (0.678 mmol, 1.00 eq) of cycloadduct (2) were dissolved in 13.5 mL of distilled methanol and 13.5 mL of 5% aqueous acetic acid were added. Then 35.1 mg (10 wt-%) of palladium on activated charcoal were added carefully under an argon-atmosphere. This suspension was hydrogenated with a balloon at room temperature for 2.5 h, while vigorous stirring. After that, the reaction mixture has been filtered through a small pad of celite and the filtrate was evaporated. The crude product was purified by column-chromatography (DCM : MeOH = 9.5 : 0.5 → 9 : 1 + 0.1% NH_3) to yield a colourless, viscid solid.

Yield: 122.4 mg, 0.389 mmol, 57%.

^{1}H-NMR (400 MHz, $CDCl_3$) δ [ppm]: 2.38 (s, 3H, N-CH_3), 3.03 - 3.17 (m, 3H, H-9 / H-10), 3.89 (s, 3H, OCH_3), 4.64 (s, 1H, H-5), 6.65 (q, $^{3}J_1$ = 8.2 Hz, $^{3}J_2$ = 25.2 Hz, 2H, H-1 / H-2).

^{13}C-NMR (100 MHz, $CDCl_3$) δ [ppm]: 21.78 (C-10), 29.98 (C-15), 32.40 (C-8), 36.66 (C-7), 43.28 (N-CH_3), 45.91 (C-16), 57.03 (OCH_3), 66.67 (C-9), 90.35 (C-5), 114.85 (C-2), 119.52 (C-1), 208.91 (C-6).

ESI-MS: 315.15 m/z $[M+H]^+$, calculated 314.16 m/z (100%).
m.p.: 98°C

1.5 Synthesis of 14β-[(*p*-nitrocinnamoyl)amino]-7,8-dihydrocodeinone (4)

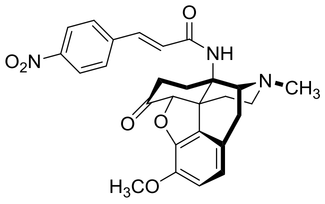

26.3 mg (0.136 mmol, 1.05 eq) of *E*-*p*-nitrocinnamic acid were dissolved in dry DMF and 54.1 mg (0.143 mmol, 1.10 eq) HBTU and 33.2 µL (0.195 mmol, 1.50 eq) of dry DIPEA were added to the solution. Then 40.8 mg (0.130 mmol, 1.00 eq) of amine (3) were transferred to the reaction mixture in dry DMF (V = 5 mL) and the solution was stirred for 18 h at room temperature. Then ethyl acetate was added and the organic phase was washed with each two portions of saturated, aqueous sodium carbonate solution, water and brine. The organic phase was dried over sodium sulphate and the crude product was subjected to column-chromatography (eluent = DCM : MeOH = 99 : 1 + 0.1% NH_3) to yield a pale yellow solid.

Yield: 55.3 mg, 0.113 mmol, 87%.

^{1}H-NMR (400 MHz, $CDCl_3$) δ [ppm]: 2.45 (s, 3H, N-CH_3), 2.92 - 3.02 (m, 1H, H-10), 3.10 (d, J = 7.3 Hz, 1H, H-9), 3.25 (d, J = 18.6 Hz, 1H, H-10), 3.89 (s, 3H, OCH_3), 4.96 (s, 1H, H-5), 6.68 - 6.78 (m, 3H, H-1 / H-2 / H-2´), 7.22 (s, 1H, NH), 7.69 - 7.73 (m, 3H, H-3´ / H-5´ / H- 9´), 8.26 (d, ^{3}J = 8.8 Hz, 2H, H-6´ / H-8´).

^{13}C-NMR (100 MHz, $CDCl_3$) δ [ppm]: 20.92 (CH_2), 30.50 (CH_2), 36.94 (C-7), 43.28 (N-CH_3), 48.02, 56.60 (C-13), 57.00 (OCH_3), 63.58 (C-9), 89.78 (C-5), 115.36 (C-2), 119.34 (C-1 / C-2´), 124.30 (C-6´ / C-8´), 125.36 (C-11), 128.65 (C-5´ / C-9´), 139.08 (C-4´), 165.77 (C-1´).

ESI-MS: 490.15 m/z $[M+H]^+$, calculated 489.19 m/z (100%).

m.p.: 270°C

1.5 Synthesis of 14β-[(*p*-nitrocinnamoyl)amino]-7,8-dihydrocodein-6β-amine (5)

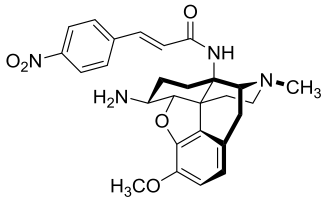

16.8 mg (0.034 mmol, 1.00 eq) of codeinone (4) were dissolved in 2 mL of methanol and 0.5 mL of tetrahydrofurane. Then 40.5 mg (0.525 mmol, 15.3 eq) of ammonium acetate and 6.7 mg (0.107 mmol, 3.12 eq) of sodium cyanoborohydride were given to the solution and the pH has been adjusted to 6 by adding some drops of 2N-HCl. This reaction was stirred for 24 h at room temperature. Then the solvents were evaporated and the crude product was taken up in chloroform. The organic phase was washed with each two portions of saturated, aqueous sodium hydrogencarbonate and water. After drying over sodium sulphate and removal of solvent, the crude product has been purified by column-chromatography (eluent = DCM : MeOH = 9.5 : 0.5 + 0.1% NH_3) to give a pale yellow solvent.

Yield: 14.2 mg, 0.029 mmol, 84%.

^{1}H-NMR (400 MHz, $CDCl_3$) δ [ppm]: 2.34 (s, 3H, N-CH_3), 3.15 (d, ^3J = 18.2 Hz, 1H, H-6), 3.86 (s, 3H, O-CH_3), 4.71 (d, ^3J = 6.9 Hz, 1H, H-5), 6.69 (dd, $^3J_1$ = 8.2 Hz, $^3J_2$ = 33.4 Hz, 2H, H-1´ / H-2´), 6.84 (d, ^3J = 15.6 Hz, 1H, H-2´), 6.98 (s, 1H, N-H), 7.59 (d, ^3J = 15.6 Hz, 1H, H-3´), 7.65 (d, ^3J = 8.6 Hz, 2H, H-5´ / H-9´), 8.21 (d, ^3J = 8.7 Hz, 2H, H-6´ / H-8´).

^{13}C-NMR (100 MHz, $CDCl_3$) δ [ppm]: 27.20 (CH_2), 29.47 (CH_2), 29.81 (CH_2), 32.04 (CH_2), 43.08 (N-CH_3), 46.25 (C-16), 56.57 (O-CH_3), 67.14 (C-9), 68.07 (C-5), 114.16 (C-2) 118.81 (C-1), 124.21 (C-6´ / C-8´), 128.46 (C-5´ / C-9´), 144.02 (C-3´), 172.95 (C-1´).

ESI-MS: 491.20 m/z [M+H]$^+$, calculated 490.22 m/z (100%).

m.p.: Decomposition > 165°C

1.6 Synthesis of 14β-[(*p*-nitrocinnamoyl)amino]-7,8-dihydrocodein-6β-tetraglycylcarboxybenzylamide (6)

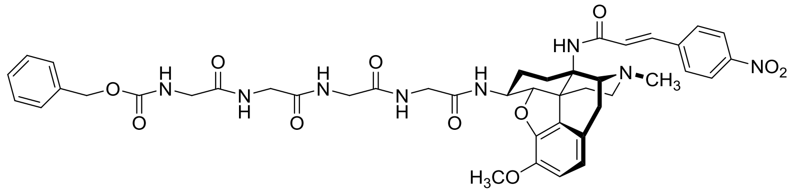

20.4 mg (53.6 µmol, 1.05 eq) of Cbz-protected tetraglycine were dissolved in 5 mL of dry DMF under an argon-atmosphere. Then 13.01 µL (76.5 µmol, 1.50 eq) of DIPEA and 21.3 mg (56.1 µmol, 1.10 eq) of HBTU were given to the solution. After that, 25 mg (51 µmol, 1.00 eq) of amine (5) were transferred to the reaction in another 5 mL of dry DMF. This solution was stirred for 18 h at room-temperature, before ethyl acetate was added and the organic phase was washed with each two portions of aqueous, saturated sodium carbonate, water and brine. After drying over sodium sulphate, the solvent was removed under reduced pressure and the crude product has been purified by column-chromatography (eluent = DCM : MeOH = 9 : 1). The product has been obtained as a white solid this way.

Yield: 39.7 mg, 46.6 µmol, 91%.

^{1}H-NMR (400 MHz, MeOD) δ [ppm]: 2.31 (t, ^4J = 7.4 Hz, 5H, N-CH_3 / H-16), 3.60 (s, 2H, Gly-CH_2), 3.65 (s, 6H, Gly-CH_2), 3.85 (s, 3H, O-CH_3), 5.08 (s, 2H, Cbz-CH_2), 6.85 (d, ^3J = 8.7 Hz, 1H, H-2), 6.91 (d, ^3J = 16.5 Hz, 1H, H-2´), 6.95 (d, ^3J = 4.8 Hz, 1H, H-1), 7.32 (d, ^3J = 6.4 Hz, 4H, Cbz-ArH), 7.66 (d, ^3J = 15.8 Hz, 1H, H-3´), 7.84 (d, ^3J = 8.7 Hz, 2H, H-5´ / H-9´), 8.28 (d, ^3J = 8.6 Hz, 2H, H-6´ / H-8´).

^{13}C-NMR (100 MHz, DMSO-d6) δ [ppm]: 22.13 (CH_2), 24.46 (CH_2), 26.60 (CH_2), 29.06 (CH_2), 43.71 (CH_2), 43.80 (N-CH_3), 51.22 (CH_2), 54.94 (O-CH_3), 79.22 (O-CH), 91.60 (CH_2), 109.82 (CH_{ar}), 122.85 (CH_{ar}), 127.78 (CH_{ar}), 127.86 (C_{ar}), 128.40 (CH_{ar}), 130.83 (CH_{ar}), 141.58 (C_{ar}), 143.86 (CH_{unsat}), 148.34 (C_{ar}), 148.53 (C_{ar}), 152.32 (C=O), 159.63 (C=O), 174.04 (C=O).

ESI-MS: 853.35 m/z $[M+H]^+$, calculated 852.34 m/z (100%).

m.p.: 167°C

1.7 Synthesis of 14β-[(*p*-nitrocinnamoyl)amino]-7,8-dihydrocodein-6β-tetraglycylamide (7)

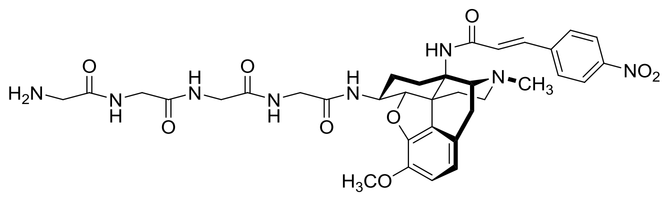

26.3 mg (30.8 µmol, 1.00 eq) of Cbz-protected compound (6) were dissolved in 3 mL of an ice-cold solution of hydrobromic acid in acetic acid (33 wt.-%). This reaction-mixture was stirred for 20 min at 0 °C, then the solvent has been removed under reduced pressure and the product has been obtained as a slight orange solid (HBr salt). It was dried in the desiccator and used without further purification.

Yield: 24.6 mg, 30.8 µmol, quant.

^{1}H-NMR (400 MHz, DMSO-d6) δ [ppm]: 2.26-2.33 (m, 5H, N-CH_3 / H-16), 3.57 (s, 6H, Gly-CH_2), 3.77 (s, 2H, Gly-CH_2), 3.78 (s, 3H, O-CH_3), 6.83 (d, ^3J = 7.1 Hz, 1H, H-2´), 6.94 (m, 2H, H-1 / H-2), 7.83 (d, ^3J = 8.8 Hz, 1H, H-3´), 7.89 (d, ^3J = 8.7 Hz, 2H, H-5´ / H-9´), 8.18 (d, ^3J = 8.7 Hz, 2H, H-6´ / H-8´).

^{13}C-NMR (100 MHz, DMSO) δ [ppm]: 19.2 (CH_2), 23.08 (CH_2), 27.1 (CH_2), 29.89 (CH_2), 42.40 (CH_2), 42.77 (N-CH_3), 45.25 (CH_2), 45.42 (CH_2), 57.99 (O-CH_3), 61.12 (CH), 63.08 (C_q), 70.34 (CH), 81.39 (CH), 108.97 (CH_{ar}), 125.48 (CH_{ar}), 131.61 (CH_{ar}), 145.71 (CH_{unsat}), 152.52 (C=O), 158.47 (C=O), 175.52 (C=O).

ESI-MS: 360.30 m/z $[M+2H]^{2+}$, 719.30 m/z $[M+H]^+$, calculated 718.31 m/z (100%).

m.p.: Decomposition > 205°C

1.8 Procedure for the labelling with Cyanine 3/5 (8/9)

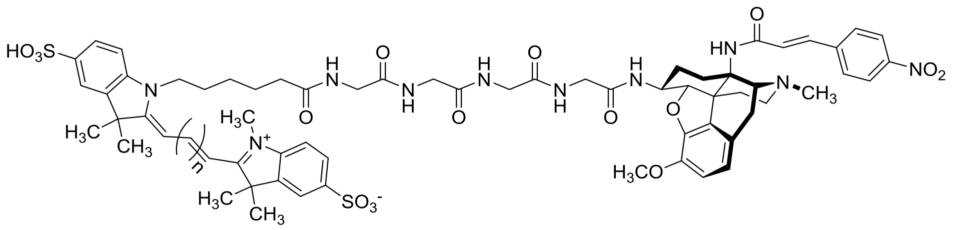

CY3: n=1
CY5: n=3

8.90 mg (12.1 µmol, 0.60 eq) of *N*-Hydroxysuccinimide-activated CY3 or 8.95 mg (12.1 µmol, 0.60 eq) of the respective CY5-analogue were dissolved in 0.50 mL of dry DMF at room-temperature under an argon-atmosphere. Then 14.5 mg (20.2 µmol, 1.00 eq) of compound (7) in 1.00 mL of dry DMF were transferred to the solution containing the respective dye. The reaction-mixture was stirred for 48 h at room-temperature and the progress was carefully monitored by TLC and LC/MS. Then the solvent

was evaporated and the crude product was directly purified by preparative HPLC on a reversed phase-column to give the target compounds (8/9) in purities of >95%.

Yield: Cpd. **8**: 6.00 mg, 4.55 µmol, 38% (CY3-labelling)
Cpd. **9**: 4.30 mg, 3.20 µmol, 26% (CY5-labelling)

ESI-MS: Cpd. **8**: 659.50 m/z $[M+2H]^{2+}$, 1318.15 m/z $[M+H]^{+}$, calculated 1317.50 m/z (100%).
Cpd. **9**: 672.50 m/z $[M+2H]^{2+}$, 1344.45 m/z $[M+H]^{+}$, calculated 1343.51 m/z (100%).

2.1 Cell culture

For the binding curves and the single-molecule experiments, Chinese Hamster Ovary (CHO) K1 (Leibniz-Institute DSMZ-German Collection of Mircoorganisms and Cell Cultures) cells were used. Cells were cultured in phenol red-free Dulbecco's modified Eagle's medium (DMEM)/F12 supplemented with 5% (v/v) FCS, 100 U/ml penicillin and 0.1 mg/ml streptomycin at 37°C and 5% CO_2. The cell lines were routinely passaged every two to three days.

2.2 Transfection

CHO-K1 cells were seeded the day before transfection at a density of 1.8 x 10^5 cells per well on ultraclean 24-mm glass coverslips in 6-well culture plates. Transfection was performed with Lipofectamine 2000 (Thermo Fisher Scientific) according to the manufacturer's protocol. For each well, 2 µg of wild-type µ-OR and 6 µl of Lipofectamine 2000 were used.

2.3 Analysis of fluorescent ligand binding by TIRF

The experiments were performed 24 h after transfection. Before imaging, each coverslip was incubated for 20 min with the indicated concentrations of either compound **8** or **9** dissolved in complete medium at 37°C, rapidly washed once with 1 ml of medium and immediately imaged in a microscopy chamber filled with 400 µl of complete medium. TIRF imaging was performed on a customised Nikon Eclipse Ti TIRF microscope using a 60x oil-immersion objective (CFI Apochromat TIRF 60x oil NA 1.49). The sample and objective were kept at 20°C via a water-cooling system.

Cells were searched and focused using a low laser power (3%) to avoid photo bleaching. Images acquired using 10% power of a 561 nm diode laser for compound 9 and 20% power of a 638 nm diode laser for compound 8 (both lasers from Coherent), were analysed using FIJI. At least 50 cells per condition from three independent experiments were used. Intensity values were normalised to those obtained with the highest concentration (10 µM). Data were fitted to a one site ligand binding model with Hill slope of 1 in Prism.

2.4 Selectivity

Fluorescent ligand binding was monitored by HTRF (Homogeneous Time-Resolved FRET). The SNAP-opioid receptors expressed at the surface of HEK293 cells were labeled with non-permanent SNAP-tag substrates derivatized with Lumi4-Tb (SNAPLumi4-Tb). Upon binding of the red fluorescent ligands on SNAP-opioid receptors, HTRF signal from the sensitized acceptor can be detected.
Dulbecco's Modified Eagle's Medium (DMEM) and fetal bovine serum (FBS) were obtained from Life Technologies (Grand Island, NY, USA). SNAP-opioid receptors plasmids and BG-Lumi4-Tb were commercialized by CisBio bioassays and provided by Dr. S. Granier.

HEK293 cells (from ATCC) were grown in DMEM supplemented with 10% FBS (without antibiotics) at 37°C, 5% CO2. Transient transfection was performed using electroporation in a volume of 200µl with 1µg SNAP-Opioid plasmids and 10 millions of HEK293 cells in electroporation buffer (50mM K2HPO4, 20mM CH3COOK and 20mM KOH, pH 7.4). After electroporation (250 V, 500µF, Bio–Rad Gene Pulser electroporator; Bio-Rad Laboratories, Hercules, CA), cells were resuspended in 10ml DMEM supplemented with 10% fetal bovine serum and seeded for 24h in a white 96-well plate (pretreated with Poly-L-Ornithine 1X) at a density of 100,000 cells per well.
24 hours after transfection, SNAP-receptors were labeled with 100nM of BG-Tb (benzylguanine-Terbium cryptate) for 1h at 37°C in Tag-Lite buffer (commercialized by CisBio bioassays). After four washing steps with PBS, the fluorescence signal from BG-Lumi4-Tb was measured on a SPARK20M plate reader (TECAN) with an excitation at 337 nm and an emission at 620 nm.
HEK293 cells expressing SNAP-opioid receptors and labeled with Lumi4-Tb were incubated with increasing concentrations of fluorescent ligands for 1h at room temperature (from 0.1 nM to 100 nM) +/- an excess of naloxone (100 µM).
HTRF signal detection was performed on a SPARK20M (TECAN). The signal was collected both at 665 nm and 620 nm. HTRF ratios were obtained by dividing the acceptor signal at 665 nm by the donor signal at 620 nm and multiplying obtained ratios by 10,000. Data were then analyzed using GraphPad Prism (GraphPad Software, Inc., San Diego, CA). Kd values of the fluorescent ligands were obtained from saturation curves of the specific binding.

2.5 Intrinsic activity

Accumulation of inositol mono phosphate (IP)

HEK-293T cells were grown to a confluence of approx. 70% and transiently co-transfected with the cDNA of the human μ-OR (gift from Ernest Gallo Clinic and Research Center, UCSF, CA) and of the hybrid G-protein $G\alpha_{qi}$ ($G\alpha_q$ protein with the last five amino acids at the C-terminus replaced by the corresponding sequence of $G\alpha_i$; gift from The J. David Gladstone Institutes, San Francisco, CA) applying the Mirus TransIT-293 transfection reagent (Peqlab, Erlangen, Germany). After one day cells were detached from the culture dish with Versene (Life Technologies, Darmstadt, Germany), seeded into black 384-well plates (10000 cells/well) (Greiner Bio-One, Frickenhausen, Germany) and maintained for 24 h at 37 °C. Agonist properties were determined by incubating compound **8** (final range of concentration from 0.1 pM up to 10 µM) in duplicates for 180 min at 37°C. Incubation was stopped by addition of the detection reagents (IP1-d2 conjugate and Anti-IP1cryptate TB conjugate each dissolved in lysis buffer) for further 60 min at room temperature. Time resolved fluorescence resonance energy transfer (HTRF) was measured using the Clariostar plate reader (BMG, Ortenberg, Germany). Data analysis was performed by nonlinear regression using the algorithms for log(agonist) vs. response of PRISM 6.0 (GraphPad, San Diego, CA) and normalization of the raw data to basal (0%) and the maximum effect of morphine (μ-OR, 100%).

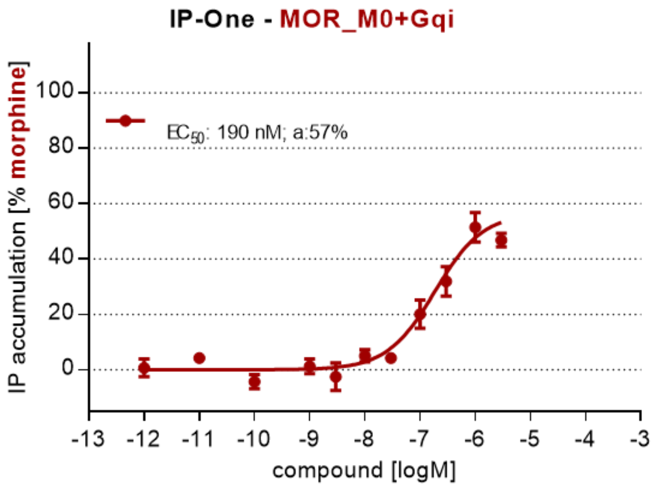

Recruitment of β-arrestin-2

HEK-293 cells stably expressing the enzyme acceptor (EA) tagged β-arrestin-2 fusion protein were transiently transfected with the ProLink tagged μ-OR-PK1, employing the Mirus TransIT-293 transfection reagent. After 24 h cells were transferred into white clear bottom 384-well plates (5000 cells/well) (Greiner Bio-One) and maintained for further 24 h at 37 °C, 5 % CO_2. To start receptor stimulated arrestin recruitment compound **8** was added to the cells to get a final concentration in a range of 1 pM to 10 μM. Incubation was continued for 90 min at 37°C. Stimulation was stopped by addition of the detection mix and further incubation for 60 min at room temperature. Chemiluminescence was determined using a Clariostar plate reader. Data analysis was done by nonlinear regression using the algorithms for log(agonist) vs. response of PRISM 6.0 (GraphPad, San Diego, CA) and normalization of the raw data to basal (0%) and the maximum effect of DAMGO (100%).

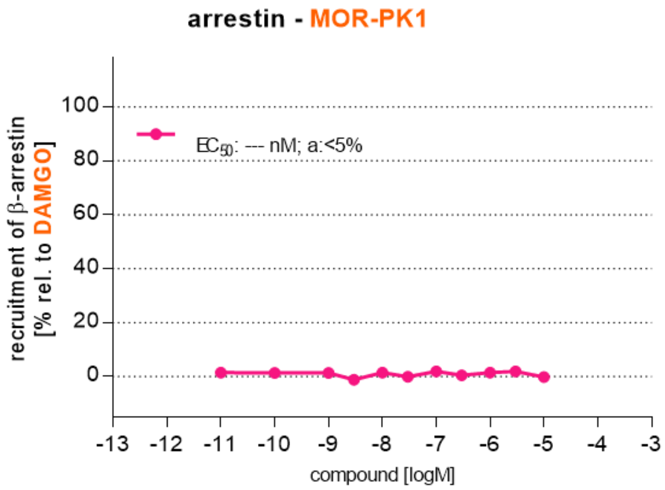

2.6 Washing experiments

Washing experiments were performed 24 h after transfection. Before imaging, each coverslip was incubated for 20 minutes at 37°C with the indicated concentration of either compound **8** or **9** dissolved in complete medium. After incubation, the coverslips were rapidly washed and immediately imaged in

a microscopy chamber filled with 400 μl of complete medium. Imaging was performed on the same customised Nikon Eclipse Ti TIRF microscope used for the analysis of fluorescent ligand binding. Images were acquired every minute, upon excitation with 15% power of either the 561 nm (compound **8**) or the 638 nm (compound **9**) diode laser, respectively. The obtained image sequences were analysed using FIJI. For each cell, a region of interest (ROI) comprising the cell was manually defined and its background-subtracted average intensity was measured. The intensity values were then normalised to corresponding ones obtained at the beginning of the image sequence. Data were plotted and fitted to a one-phase exponential decay in Prism 6, GraphPad Software Inc.. Photo bleaching during image acquisition was negligible, as verified by control experiments in which the same number of frames were acquired very rapidly (within 600 ms) showing less than 2% reduction of the fluorescent signal at the end of the acquisition for Cy3. For the compound bound to Cy5 the bleaching was significantly higher with 21%. Therefore the washing data for Cy5 were corrected for the bleaching.

2.7 Single-molecule experiments

Five hours after transfection, the cells were incubated for 20 minutes with 1 μM of compound **9** dissolved in complete medium. After incubation, the coverslips were rapidly washed and immediately imaged in a microscopy chamber filled with 400 μl of complete medium. Imaging was performed on the same customised Nikon Eclipse Ti TIRF microscope used for the analysis of fluorescent ligand binding, but this time with a 100x oil-immersion objective (CFI Apo TIRF 100x, NA 1.49).

The sample was searched and focused using low laser power (3%) of a 561 nm diode laser. Movies of 200 frames were recorded with 15% laser power in crop mode with an acquisition rate of one image every 28.4 ms, resulting in 35 frames per second. The acquisition of movies was performed only in the first four to five minutes after mounting. These conditions result in less than 40% dissociation of either ligand from the receptor, as estimated by the washing experiments.

For the two-colour single-molecule experiments, the cells were incubated for 20 minutes with a mixture of 1 μM compound 9 (Cy3) and 0.5 μM compound 8 (Cy5) in order to obtain similar labelling efficiencies. As a negative control, cells were co-transfected with wild-type μ-OR and CD86 with a N-terminal SNAP-tag. After 5 hours of transfection, cells were incubated with 1 μM Caco-Gly4-Cy3 and 1 μM SNAP Alexa Fluorophore 647 (NEB). The settings for data acquisition were the same as above.

To check if colocalisations happen at clathrin-coated pits, control experiments were performed by co-expression of Clathrin–GFP. Movies of μ-OR – μ-OR with compound 8 and 9 were acquired as mentioned above. Immediately after, a movie of 200 frames of Clathrin-GFP was obtained using 30% power of a 488 nm laser (Coherent).

2.8 Analysis of single-molecule experiments

Single-molecule movies were selected for low receptor density with in average of 0.85 receptors per μm^2, and a mask corresponding to the cell shape was applied to perform automated single-particle detection and tracking in Matlab using u-track software (1) as described in (2). Single-particles were located by fitting 2D Gaussian functions to the diffraction-limited spots taking into account the point spread function of the microscope. Next the detected particles were linked in consecutive frames. Therefore costs were assigned for every potential event like blinking, merging, splitting and leaving out the focal plane. The overall tracking with the lowest costs was thus selected. The obtained single trajectories were then analysed for their time-averaged mean square displacement (TAMSD), which was used to categorise the particles into four classes of motion: virtually immobile, sub-diffusive, diffusive or super-diffusive motion. (2-4) The four classes were separated based on D, their diffusion

coefficient and α, the anomalous diffusion coefficient, whereas virtually immobile (D < 0.01 $\mu m^2 s^{-1}$), sub-diffusion ($\alpha < 0.75$), normal diffusion ($0.75 \leq \alpha \leq 1.25$) and super- diffusion ($\alpha > 1.25$).

The two-colour experiments were corrected for distortion and misalignment of the detectors using fluorescent beads. Both channels were analysed as described above to obtain single-particle trajectories. As a control for random colocalisation, movies with labelled CD86 and μ-OR were analysed in the same way as the movies with μ-OR labelled with compound 9 and 8. The obtained trajectories for the two colours were further analysed for colocalisations. A search radius R_0=265nm was defined and each particle in channel two, falling into this R_0 for a particle in channel 1 was registered as a colocalisation. This was done for each frame and thus colocalisation could be linked analogous to the single-particle tracking taking. Overall a starting and terminating frame was obtained for each colocalisation thus the probability density function of colocalisation times could be computed. Those interaction times were further analysed comparing those from the μ-OR – μ-OR interactions with those from μ-OR – CD86. In order to isolate the true interaction times from the colocalisation times that consist in the true interactions durations plus random ones, a deconvolution was applied using the Lucy-Richardson algorithm. (5) The colocalisations observed between CD86 and μ-OR were considered as a control for random colocalisations. By deconvolving the μ-OR – μ-OR colocalisation times with the colocalisation times from μ-OR – CD86, the proportion of "true" interactions (F_{true}) was deduced. For more details, see Supplementary Methods of (6).

The deconvolved data was normalised and the fraction of surviving interactions after time t, noted $F_r(t)$, was fitted to an exponential decay function

$$F_r(t) = F_{true} e^{-(k_{off}+k_{loss})t}.$$

The quantity k_{loss} accounts for premature termination due to photo-bleaching or particle loss from errors in detection and tracking. This was obtained previously from control experiments. Thus the dissociation rate k_{off} was derived from the deconvolved colocalisation data.

The quantity k_{on} was calculated from F_{true} and the rate of new colocalisations per unit of area $d[D]_\rho/dt$ with the following equation

$$\frac{d[D]_\rho}{dt} \cdot F_{true} = k_{on}[\mu_1]_\rho[\mu_2]_\rho,$$

whereas $[\mu_1]_\rho$ and $[\mu_2]_\rho$ are the densities of monomers in each channel estimated as the difference between the measured densities $[\mu_1]_\rho$ and $[\mu_2]_\rho$ in channel 1 and 2 and the estimated concentration of dimers such that $[\mu_1]_\rho = [Ch1]_\rho - [D]_\rho$ and $[\mu_2]_\rho = [Ch2]_\rho - [D]_\rho$.

The density of dimers $[D]_\rho$ was deduced thanks to the equality between the rate of association and the rate of dissociation through the formula

$$[D]_\rho = \frac{1}{k_{off}} \frac{d[D]_\rho}{dt} \cdot F_{true},$$

such that k_{on} could be expressed as a function of measurable quantities

$$k_{on} = \frac{\frac{d[D]_\rho}{dt} \cdot F_{true}}{\left([Ch1]_\rho - \frac{1}{k_{off}} \frac{d[D]_\rho}{dt} \cdot F_{true}\right)\left([Ch2]_\rho - \frac{1}{k_{off}} \frac{d[D]_\rho}{dt} \cdot F_{true}\right)}.$$

An estimation of k_{on} has been done between each successive frames and the presented k_{on} was obtained by averaging the obtained k_{on} over time.

The obtained interactions were further analysed for colocalisation with clathrin-coated pits (CCPs). Additional to the two channels with μ-OR's a third channel was measured with GFP-clathrin, which images were stacked to check for colocalisation with interactions that lasted longer than 20 frames. Therefore the CCPs were localised by fitting a 2-D Gaussian, also giving the estimated width L of the pits. An interaction was defined as 'inside a CCP' when one of the colocalising particles was localised once during the interaction within L of one CCP. If the colocalising particles were not within less than L of any CCP, then the colocalisation was considered as 'outside CCPs'.

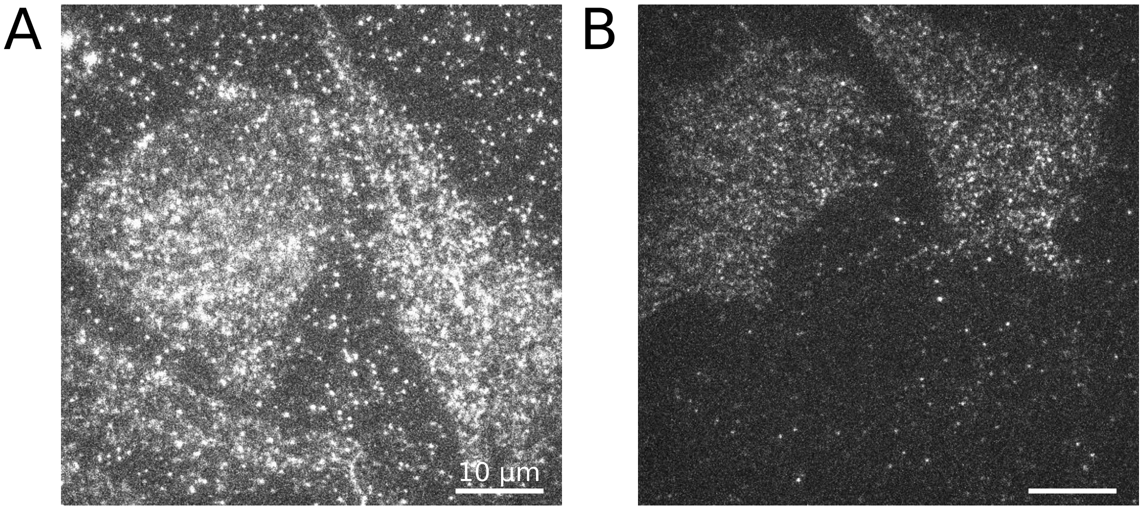

Supplementary figure 1: Comparison of different linker. A shows CACO linked to Cy3 via an alkyne chain. While cells expressing μOR are clearly distinguishable the compound additionally stick to the coverslip resulting in high background. The introduction of a tetraglycine as linker (compound 8), shown in B, improved the signal-to-noise ratio significantly.

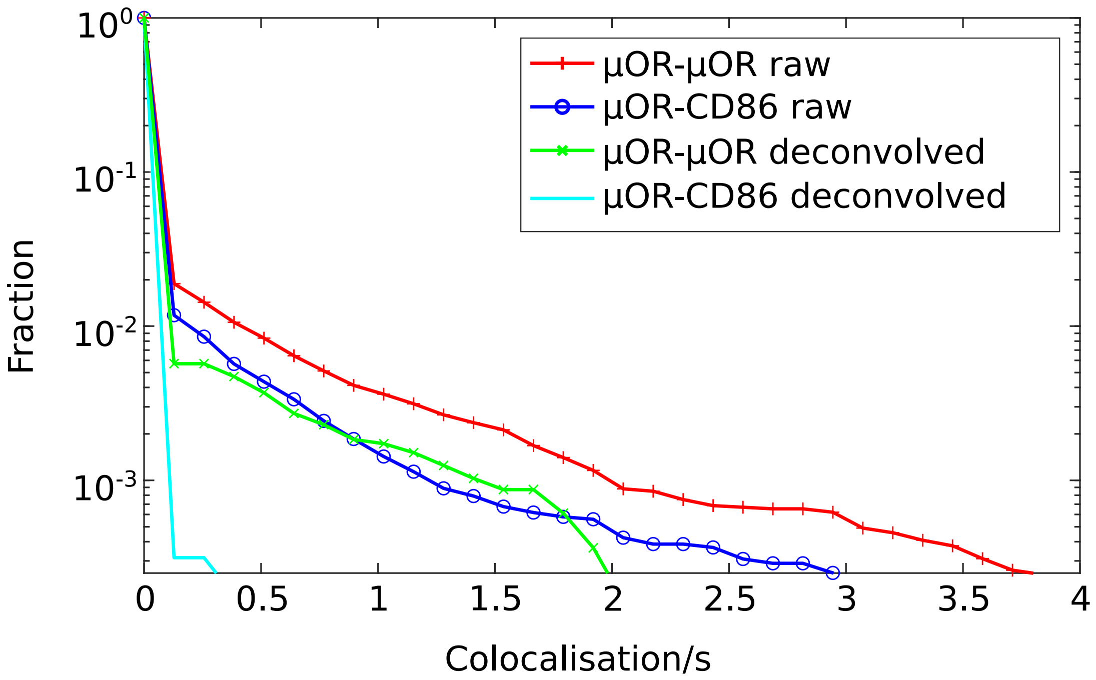

Supplementary figure 2: Deconvolution of μOR and CD86 interactions. The observed co-localisation times of μORs with each other (red) and co-localisation times between μOR and CD86 (blue). The deconvolved co-localisation times for μOR interactions are shown in green. The deconvolution of the control is shown in cyan and is expected to show a single peak at zero and decrease fast. Data are from 51832 co-localisations obtained from 25 individual cells for μOR- μOR and 61259 colocalisations for CD86- μOR.

References

[1] K. Jaqaman, D. Loerke, M. Mettlen, H. Kuwata, S. Grinstein, S. L. Schmid, G. Danuser, *Nat. Methods* **2008**, *5*, 695-702.

[2] R. Metzler, J. H. Jeon, A. G. Cherstvy, E. Barkai, *Phys. Chem. Chem. Phys.* **2014**, *16*, 24128-24164

[3] A. Andreanov, D. S. Grebenkov, *J. Stat. Mech. Theor. Exp.* **2012**, P07001.

[4] Y. Lanoiselée, G. Sikora, A. Grzsesiek, D. S. Grebenkov, A. Wyłomańska *Phys. Rev. E* **2018**, *98*, 062139.

[5] L. B. Lucy, *Astron. J.* **1974**, *79*, 745-754.

[6] T. Sungkaworn, M.-L. Jobin, K. Burnecki, A. Weron, M. J. Lohse, D. Calebiro, *Nature* **2017**, *550*, 543-547.
